# Association of the Epithelial–Mesenchymal Transition (EMT) with Cisplatin Resistance

**DOI:** 10.3390/ijms21114002

**Published:** 2020-06-03

**Authors:** Milad Ashrafizadeh, Ali Zarrabi, Kiavash Hushmandi, Mahshad Kalantari, Reza Mohammadinejad, Tahereh Javaheri, Gautam Sethi

**Affiliations:** 1 Department of Basic Science, Faculty of Veterinary Medicine, University of Tabriz, Tabriz 5166616471, Iran; dvm.milad1994@gmail.com; 2 Sabanci University Nanotechnology Research and Application Center (SUNUM), Tuzla, Istanbul 34956, Turkey; alizarrabi@sabanciuniv.edu; 3 Center of Excellence for Functional Surfaces and Interfaces (EFSUN), Faculty of Engineering and Natural Sciences, Sabanci University, Tuzla, Istanbul 34956, Turkey; 4 Department of Food Hygiene and Quality Control, Division of Epidemiology, Faculty of Veterinary Medicine, University of Tehran, Tehran 1417414418, Iran; houshmandi.kia7@ut.ac.ir; 5 Kazerun Health Technology Incubator, Shiraz University of Medical Sciences, Shiraz 1433671348, Iran; 6 Department of Genetic Science, Tehran Medical Science Branch, Islamic Azad University, Tehran 19168931813, Iran; Mahshadk73@yahoo.com; 7 Pharmaceutics Research Center, Institute of Neuropharmacology, Kerman University of Medical Sciences, Kerman 1355576169, Iran; 8 Health Informatics Lab, Metropolitan College, Boston University, Boston, MA 02215, USA; 9 Department of Pharmacology, Yong Loo Lin School of Medicine, National University of Singapore, Singapore 117600, Singapore; phcgs@nus.edu.sg

**Keywords:** cisplatin, cancer therapy, chemoresistance, epithelial–mesenchymal transition (EMT), signal transduction

## Abstract

Therapy resistance is a characteristic of cancer cells that significantly reduces the effectiveness of drugs. Despite the popularity of cisplatin (CP) as a chemotherapeutic agent, which is widely used in the treatment of various types of cancer, resistance of cancer cells to CP chemotherapy has been extensively observed. Among various reported mechanism(s), the epithelial–mesenchymal transition (EMT) process can significantly contribute to chemoresistance by converting the motionless epithelial cells into mobile mesenchymal cells and altering cell–cell adhesion as well as the cellular extracellular matrix, leading to invasion of tumor cells. By analyzing the impact of the different molecular pathways such as microRNAs, long non-coding RNAs, nuclear factor-κB (NF-ĸB), phosphoinositide 3-kinase-related protein kinase (PI3K)/Akt, mammalian target rapamycin (mTOR), and Wnt, which play an important role in resistance exhibited to CP therapy, we first give an introduction about the EMT mechanism and its role in drug resistance. We then focus specifically on the molecular pathways involved in drug resistance and the pharmacological strategies that can be used to mitigate this resistance. Overall, we highlight the various targeted signaling pathways that could be considered in future studies to pave the way for the inhibition of EMT-mediated resistance displayed by tumor cells in response to CP exposure.

## 1. Introduction

Cancer is still an increasing challenge for public health and is the second leading cause of death worldwide [1]. The newest statistics related to the incidence rate of cancer in the United States demonstrate that each day, 4950 people are diagnosed with cancer and its annual incidence rate is 1,806,590. The common cancers are different in men and women. Prostate, lung, and colorectal cancers are among the most common cancers in males, while breast, lung, and colorectal cancers are prevalent in females. On the other hand, due to enhanced life expectancy, the population of the world is going to be older in the following years. For instance, in United States, the population of aged people is suggested to be 19 million in 2060, which is significantly higher compared to the 6.6 million aged people in 2016 [2]. This is related to the fact that the possibility of cancer development is higher in aged people. In addition, it is less likely to diagnose aged patients with cancer at early and local stages [3]. These statements are in agreement with the fact that dealing with cancer is of the utmost importance in the modern world and it demands extensive research into finding novel treatments for this life threatening disorder [4,5,6,7,8,9,10,11].

Currently, regardless of diagnostic methods, a number of treatment strategies can be applied for cancer therapy. Chemotherapy is a first-line treatment for cancer due to its minimally invasive nature and satisfactory results in clinical trials [12]. However, patients with cancer and physicians have faced a growing difficulty in chemotherapy, known as chemoresistance [13]. Studies have shown that frequent application of chemotherapeutic agents with high doses has led to the emergence of chemoresistance. After the appearance of chemoresistance, research was performed to discover new chemotherapeutic agents to improve chemotherapy efficacy. Very soon, it was found that cancer cells develop resistance to chemotherapeutic agents by switching between molecular pathways and mechanisms to ensure their proliferation and malignancy [14]. Currently, the focus has been directed towards using anti-tumor drugs along with chemotherapeutic agents, in addition to gene therapy [15,16]. Finding an effective regimen for inhibition of chemoresistance in cancer therapy relies on revealing the molecular pathways and mechanisms involved in development of resistance of cancer cells to chemotherapy. To date, thanks to extensive research in the field of chemoresistance, numerous targets have been explored for chemoresistance/chemosensitivity [17,18,19,20].

In the present review, we investigate the role of epithelial–mesenchymal transition (EMT) in the emergence of cisplatin (CP) resistance. CP is a key member of platinum compounds with excellent anti-tumor activity against different cancers such as breast cancer, prostate cancer, bladder cancer, lung cancer, brain tumors, and so on [21,22,23]. The rational reasons for selection of CP are as follows: (1) its anti-tumor activity has been extensively examined, particularly in clinical trials [24,25,26], and (2) the molecular mechanisms and pathways involved in CP resistance/sensitivity have been explored [27,28,29]. Consequently, it has been possible to implicate the process of EMT in drug resistance and directing further studies towards targeting EMT in suppressing resistance developed against CP treatment.

## 2. Cisplatin in Cancer Therapy

Chemotherapy is still one of the most common modalities used in cancer therapy [30]. A variety of chemical agents, such as alkylating compounds and antimetabolites, have been applied in cancer therapy to suppress the progression and invasion of cancer cells by affecting different hallmarks of cancer cells [31]. More recently, platinum-based drugs have attracted much attention in the treatment of malignant tumors because of their effectiveness in targeting different signaling pathways and mechanisms that control cancer progression [32]. CP is one of the most common types of platinum-based medicines, which has shown great potential in the treatment of several types of cancer, including brain tumors [33,34], lung cancer [35], breast cancer [36], bladder cancer [37], prostate cancer [38], oral cancer [39], and neck cancer [40]. After the discovery of this anti-tumor agent in 1978, its application has undergone a tremendous increase [41,42]. A very good example of its effectiveness has been shown when combined with Clarithromycin to suppress the invasion and proliferation of ovarian cancer cells, which elevates apoptotic cell death by increasing the production of reactive oxygen species (ROS) [43]. A combination of CP and a 5-aminopyrazole derivative lead compound (BC-7) stimulates M-phase arrest and prevents the proliferation of cervical cancer cells [44]. It is worth mentioning that CP and docetaxel (DOX) can be administered prior to surgery to increase the survival resection rate, reduce intraoperative blood loss, and extend the patient’s survival time. Chemotherapy and radiotherapy can be used simultaneously (CP, DOX, and 5-fluorouracil) to effectively treat advanced esophageal cancer [45].

Nanoparticles (NPs) are promising candidates for the targeted administration of CP. The use of multifunctional NPs may also improve the anti-tumor activity of CP [46]. It has been shown that NPs offer the possibility of administering CP together with other anti-tumor agents to increase the number of cells undergoing apoptosis [47]. It has been proposed that CP induces apoptotic cell death through the mitochondrial signaling pathway and activation of the ROS/AMP-activated protein kinase signaling pathway (AMPK). Administration of β-elemene in combination with CP enhances the effect on the ROS/AMPK axis and consequently improves anti-tumor activity [48]. These studies show that CP is effective in inhibiting the growth and proliferation of cancer cells. Overall, CP induces DNA damage in cancer cells and suppresses cancer cell proliferation and viability. The combination of CP with other chemotherapeutic agents has shown great potential in cancer therapy [49,50,51,52,53,54].

**Figure 1:**
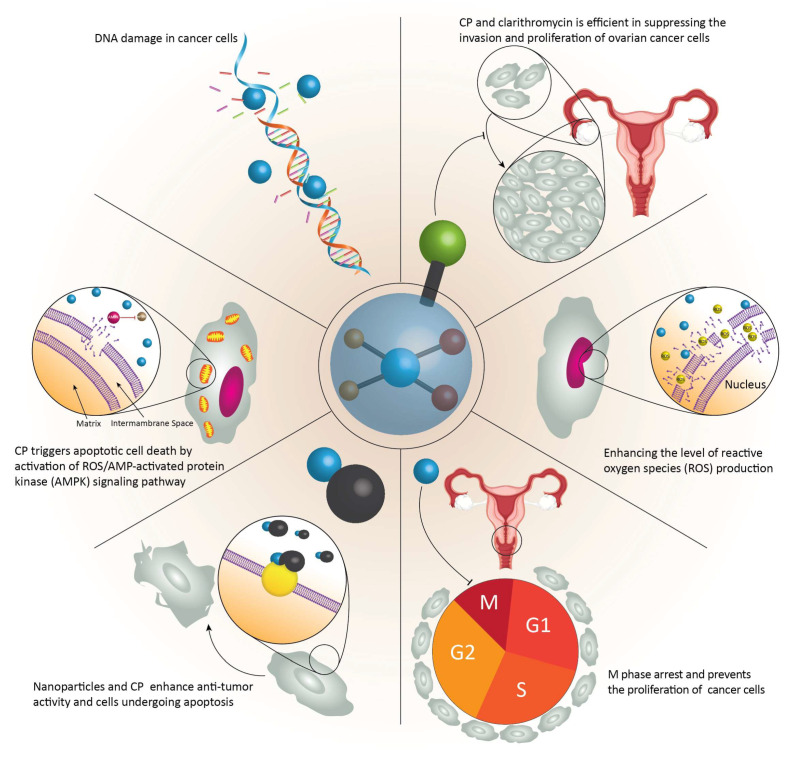
The involvement of different molecular pathways in anticancer actions of cisplatin (CP).

However, despite its great potential in the treatment of cancer, the use of CP in cancer therapy has a number of disadvantages [55]. One of these is adverse effects on the body’s organs and systems resulting from increased production of ROS and stimulation of mitochondrial dysfunction [56,57,58,59]. Gastrointestinal disorders, ototoxicity, neurotoxicity and hepatotoxicity are some of the toxic effects of CP [60]. Regardless of the undesirable effects of CP, which can be mitigated by antioxidant compounds [61,62,63], the main difficulty with CP therapy is the resistance developed by cancer cells against this drug. This could be due to the use of dynamic and flexible molecular pathways by cancer cells to improve their proliferation and survival [64,65,66,67]. It has been shown that frequent use of CP at high doses is associated with the reduced effectiveness of CP in suppressing invasion and malignancy of tumor cells [68,69]. A number of reasons have been attributed for this issue and in this paper, we have focussed on the role of EMT in the development of resistance to CP treatment (Figure 1).

## 3. Cisplatin Resistance

Recently published articles have shed some light on the molecular pathways and mechanisms involved in CP resistance. A number of strategies have been applied to enhance the sensitivity of cancer cells in CP therapy. Autophagy is a degradation process that serves to reduce stress and maintain proper cell function under physiological conditions [70,71,72]. This mechanism has a controversial function in cancer; on one hand autophagy in cancer therapy suppresses the viability and proliferation of cancer cells [73,74]. However, studies have shown that autophagy may be involved in increasing the survival and propagation of cancer cells, and can also mediate resistance to chemotherapy [75,76,77,78]. The long non-coding RNA (lncRNA) SNHG14 is able to induce CP resistance in colorectal cancer cells by inducing autophagy activation via ATG14 upregulation [79]. The autophagy mechanism also prevents the repolarization of tumor-associated macrophages (TAMs) to stimulate chemoresistance against CP therapy [80].

A number of strategies have been used to increase the sensitivity of cancer cells to CP therapy. To suppress autophagy during CP therapy, propofol was administered as an anti-tumor agent [81] in combination with CP to inhibit autophagy by downregulating the MALAT1/microRNA (miR)-30e/ATG5 axis, resulting in sensitivity of gastric cancer cells (GC cells) to CP therapy [82]. Oridonin supplementation can suppress autophagy by beclin-1 downregulation to increase the efficacy of CP therapy in ovarian cancer cells [83]. In addition, upregulation of miR-29c-3p and miR-138-5p inhibits autophagy and sensitizes cancer cells to CP-induced autophagy [84,85]. It is worth mentioning that the involvement of autophagy in CP resistance has been controversial. It was mentioned that stimulation of autophagy may be associated with CP resistance. However, there are a number of studies, which indicate that induction of autophagy can suppress CP resistance. For example, arctigenin sensitizes colon cancer cells to CP therapy by autophagy induction [86]. These studies underline the fact that the autophagy mechanism plays a crucial role in CP resistance. It appears that the involvement of autophagy in CP resistance varies between different cancer cells (context dependent).

In addition to autophagy, miRs may contribute to resistance to CP therapy. miRs are short non-coding RNAs that are able to regulate vital biological mechanisms such as apoptosis and differentiation [87,88,89]. The potential role of miRs in regulating the migration and invasion of cancer cells has been demonstrated [90,91]. In particular, lncRNAs can act as upstream modulators of miRs [92,93]. Thus, there is a close relationship between lncRNAs, miRs, and CP resistance. The newly published articles have also shown the involvement of miRs and lncRNAs in CP resistance. In oral squamous cell carcinoma (OSCC), the expression of miR-132 is downregulated, which mediates the resistance of OSCC to CP therapy [94]. The downregulation of miR-182-5p induces resistance of GC cells to CP therapy [95]. Notably, OSCC reduces the expression of miR-26b to inhibit CP-mediated DNA damage and induce resistance to chemotherapy [96]. LncRNAs are also involved in CP therapy, and by amplification/reduction of their expression it is possible to reduce the viability and proliferation of cancer cells and sensitize them to CP therapy [97,98,99]. Other molecular signaling pathways such as the Wnt signaling pathway [38,100], phosphoinositide 3-kinase-related protein kinase (PI3K) [77], nuclear factor erythroid 2-related factor 2 (Nrf2) [101], and sirtuin 1 (SIRT1) [102] may also be involved in the resistance of cancer cells to CP therapy. These studies show that CP resistance is a common phenomenon that is due to the increased use of CP in cancer therapy. Interestingly, various genetic strategies have been used to modify the expression of genes involved in CP resistance and increase the effectiveness of this chemotherapeutic agent. In addition, pharmacologically stimulating the sensitization of cancer cells by other chemosensitizers can also be important for increasing the efficacy of CP therapy.

## 4. EMT Process in Healthy and Cancerous Tissues

EMT is a physiological process necessary for embryonic development and is involved in mesoderm formation and neural crest removal [92,103,104,105]. This highly conserved mechanism is able to control morphogenesis in other cells [104,106]. Cancer cells apply EMT mechanism to enhance their migration and invasion, and consequently, ensure their survival and malignancy [107,108,109]. The EMT mechanism is reversible and can be regulated via a variety of molecular signals such as lncRNA, miR, Akt, and PI3K signaling pathways [110,111,112,113,114]. Both normal and cancer epithelial cells can use the EMT mechanism in the way of elevating their migratory ability and diffusion into neighboring tissues [115,116,117]. During EMT mechanisms, stationary epithelial cells are transformed into mobile mesenchymal cells [118,119]. In EMT, a spindle-like morphology is acquired, cell–cell adhesions are disrupted, and the polarity is changed from apical–basal polarity into front-end–back-end polarity [76,120]. Two proteins known as E-cadherin and N-cadherin are suggested to play a significant role in EMT mechanisms [121,122]. The cadherin proteins shape a cadherin–catenin adhesion complex by attachment into β- and α-catenin via their cytoplasmic tails [123].

Increasing evidence demonstrates the possible tumor suppressor function of E-cadherin and its upregulation in normal epithelial cells [123,124]. In non-cancerous epithelial cells, E-cadherin preserves epithelial phenotype and tissue homeostasis via targeting various molecular pathways. The EMT is an important mechanism for physiological processes such as embryogenesis, wound healing, fibrosis, and so on. Thus, modulation of EMT can also be of importance in treatment of non-cancerous diseases [125,126,127]. It has been shown that E-cadherin downregulation may be associated with the development of a number of cancers such as breast cancer [128], lung cancer [129], skin cancer [130], and GC [131]. This can be related to the EMT mechanism. It is held that a decrease in E-cadherin triggers the EMT mechanism, leading to an increased invasion and migration of tumor cells [132]. A variety of studies have revealed that E-cadherin upregulation may be associated with less migratory ability of cancer cells and their sensitivity to cell death that can be attributed to the EMT mechanism inhibition [133,134,135,136]. These studies highlight the fact that during EMT mechanism, a decrease occurs in E-cadherin protein level to ensure the metastasis and invasion of tumor cells. The story for N-cadherin is completely different. The N-cadherin protein is at the minimum level in non-cancerous epithelial cells, whereas its abundance is evident in cancer epithelial cells [137]. The upregulation of N-cadherin in normal epithelial cells indicates an imminent EMT and development of cancer [138]. N-cadherin contributes to the metastasis and dissemination of epithelial cells from other cells during EMT mechanism [139,140]. Thus, it is conspicuous that N-cadherin and E-cadherin proteins undergo upregulation/downregulation during EMT mechanism, respectively [141,142].

The EMT mechanism is involved in the transformation of static epithelial cells into mobile mesenchymal cells. This mechanism is of crucial importance for the metastasis and migration of tumor cells. There are a number of interactions between the molecular pathways and the EMT mechanism to promote the invasion of cancer cells. The Musashi RNA binding protein 2 (MSI2) contributes to cell differentiation and regulates stem cells and asymmetric division [143]. The collected data show that abnormal expression of MSI2 is associated with the development of cancer [144,145,146]. In pancreatic cancer cells, MSI2 triggers the zinc-finger E-box binding homeobox (ZEB)1/ERK/MAPK axis to increase EMT in these cancer cells, resulting in increased migration and invasion [147]. It appears that miR-1228 increases the proliferation and metastasis of ovarian cancer cells by EMT induction [147].

Four and a half LIM domain proteins (FHL) are evolutionary conserved domains involved in the interaction with molecular pathways such as Wnt [148], Ras [149], and tumor growth factor (TGF) [150], to modulate the growth and invasion of cancer cells. FHL proteins have been shown to regulate EMT in cancer cells [151]. By preventing the ubiquitination of Snail 1 and Twist1, FHL3 stimulates EMT to ensure the malignancy of pancreatic cancer cells [152]. Yes-associated protein 1 (YAP1) and tafazzin (TAZ1) are factors involved in the initiation of cancer development [153]. These proteins exert different strategies in the development of cancer and promoting cancer rigidity. In a recently published article, it was found that stimulation of YAP1/TAZ1 translocation is associated with increased viability of colorectal cancer cells (CRCs). YAP1 and TAZ1 are the downstream mediators of the LINCO1413/hnRNP-k/ZEB1 axis and their induction facilitates the EMT mechanism [154].

The ubiquitin-conjugating enzyme E2O (UBE2O) is expressed in tissues such as brain, heart, liver, and muscle [155]. This protein has several vital functions and its association with the EMT mechanism is of interest. In breast cancer cells, UBE2O degrades AMPK2 by enhancing its ubiquitination and paves the way for the activation of the mammalian target rapamycin (mTOR). This axis increases the proliferation and metastasis of breast cancer cells by inducing the EMT mechanism [156]. MiR-4472 is involved in chemoresistance and cancer development [157]. The study of the molecular pathways has shown that the EMT mechanism can be induced by miR-4472 during cancer progression [158]. Overall, stimulation of the EMT mechanism is a positive factor in tumor metastasis and migration, while it is associated with the poor prognosis of cancer patients [159,160].

EMT can contribute to the resistance of tumor cells to chemotherapy. Afatinib is an anti-tumor agent of a second-generation epidermal growth factor receptor (EGFR) tyrosine kinase inhibitor, which is commonly used to treat lung cancer [161,162]. Lung cancer cells may use the EMT mechanism to enhance their migration and malignancy and to achieve resistance to afatinib. To provide effective treatment for lung cancer, we should use a combination of afatinib and EMT targets such as histone deacetylase inhibitors [163]. In fact, another study emphasized that downward regulation of Twist1 can be seen as a promising strategy to sensitize lung cancer cells to EMT-induced chemotherapy-induced resistance [164]. Small molecule inhibitors targeting FGFR1 are widely used in cancer treatment. AZD4547 is one such molecule with the ability to reduce the viability and proliferation of cancer cells. EMT induction has significantly reduced the effectiveness of AZD4547 in cancer therapy [165]. In addition, miRs can significantly contribute to the activation of EMT and chemoresistance. Paclitaxel (PTX) is a chemotherapeutic agent discovered in 1963, isolated from the bark of the Pacific yew *Taxus brerifolia* [166]. This compound has shown great potential in the treatment of various types of cancer [167]. In recent years, resistance to PTX has been a common phenomenon. It is believed that an increase in the expression of miR-181a induces the EMT mechanism and mediates resistance of ovarian cancer cells to PTX therapy [168]. Overall, the studies confirm that the EMT mechanism is not only crucial for the progression and malignancy of cancer cells, but also induces resistance to chemotherapy and reduces apoptotic cell death [169,170,171,172].

## 5. Cisplatin Induces EMT-Mediated Cancer Chemoresistance

TAMs are one of the main infiltrations of immune cells into the microenvironment of the tumor and they interact with solid tumors since they are involved in the metastasis of cancer cells [173,174,175,176]. Classically activated macrophages (CAMs) and alternatively activated macrophages are two main types of TAMs [177]. In particular, CAMs appear to promote the migration and malignancy of cancer cells such as hepatocellular carcinoma (HCC), ovarian, and oral cancers [178,179,180]. Chemotherapy with CP is associated with an increase in the migration ability of CAMs. The study of molecular markers shows that the induction of CAMs by CP triggers the EMT mechanism. It is held that CP just stimulates CAMs to secrete chemokine ligand 20 (CCL20) without affecting their phenotype [181]. The chemokine ligand 20 (CCL20) is able to recruit T helper cells to maintain the immunosuppressive microenvironment and ensure the progression of the cancer [182,183,184]. The chemokine receptor 6 (CCR6) is a secondary target of CCL20 that induces cancer migration and metastasis [185]. Interestingly, chemotherapy with CP stimulates CAMs to secrete CCL20, then the CCL20/CCR6 axis enhances tumor cell migration and induces the EMT mechanism, thereby leading to EMT-mediated drug resistance [181].

It appears that not a single factor is responsible for the resistance of cancer cells to CP chemotherapy and a number of diverse mechanism(s) may be involved (summarized in Table 1). The ataxia telangiectasia mutated (ATM) is a key member of phosphoinositide 3-kinase-related protein kinase (PI3K) family, which participates in DNA damage response. Endogenous factors such as ROS and exogenous factors including irradiation are able to induce ATM activation. ATM can subsequently trigger cell cycle checkpoint machinery, DNA repair or apoptosis in response to the aforementioned stimuli [186,187]. On the other hand, Schlafen 11 (SLFN11) is an onco-suppressor factor that enhances sensitivity of cancer cells into anti-tumor agents [188]. Both ATM upregulation and SLFN11 downregulation can activate EMT to stimulate tumor cells’ resistance to CP [189]. CP is also able to increase EMT markers such as Snail to reduce the sensitivity of tumor cells and ensure their migration and metastasis [190]. Although high doses of CP over a long period could induce CP resistance, an experiment conducted by Liu and colleagues showed that short and low concentrations of CP via affecting the EMT can also induce resistance in tumor cells [191]. In addition, CP induces EMT via the activation of oncogenic NF-κB signaling pathway [192]. The discovery of the underlying molecular signaling pathway may therefore pave the way for more targeted influencing and increasing the efficacy of CP in chemotherapy.

Autophagy is considered as a highly conserved mechanism when cells have to degrade the additional toxic and aged organelles and macromolecules [193,194]. The role of autophagy in cancer still remains unclear [195,196]. It appears that the involvement of autophagy in cancer progression/inhibition depends on the context and the type of cancer [197,198]. Recently published articles have shown that autophagy can function as a cytoprotective mechanism to improve the viability and proliferation of tumor cells and induce resistance to chemotherapy [199,200,201,202]. The same applies to CP. It was noted that the administration of CP is associated with the induction of autophagy. After activation of autophagy, the migration of cancer cells is upregulated, and the cancer cells are able to acquire EMT (enhanced levels of vimentin). Inhibition of autophagy is believed to interfere with the EMT mechanism and reduce the malignancy and invasion of tumor cells [203]. Therefore, this aspect of autophagy should be considered in chemotherapy.

## 6. Strategies to Attenuate EMT-Related Cisplatin Resistance

### 6.1. Cluster of Differentiation Role

CD13 is a transmembrane glycoprotein with the ability to activate tumor angiogenesis and adhesion [204]. The upregulation of CD13 is associated with the poor prognosis of GC [205]. On the other hand, the epithelial membrane protein 3 (EMP3) is a peripheral myelin protein whose overexpression can increase the invasive ability of cancer cells [206,207]. There is a close relationship between CD13, EMP3, EMT, and CP resistance, so that increased expression of CD13 increases the expression of EMP3 to stimulate the PI3K/Akt/NF-κB axis. This leads to induction of the EMT mechanism and resistance of GC cells to apoptosis and inhibitory effects of CP. Ubenimex is an inhibitor of CD13 that suppresses the signaling pathway mediated by CD13 to inhibit CP resistance by downregulating EMT. Mechanistically, ubenimex enhances CpG island hypermethylation for which CD13 inhibition may be needed. Subsequently, a disruption occurs in downstream signaling pathway PI3K/Akt/NF-κB that suppresses both autophagy and EMT to sensitize cancer cells in CP chemotherapy [208].

### 6.2. The Contribution of Exosomes

Exosomes are small membrane vesicles that are able to promote the progression and metastasis of tumor cells [209,210]. It seems that exosomes are involved in drug resistance through the transmission of lncRNAs [211,212]. Exosomal transfer of lncRNA HOTTIP (an oncogenic lncRNA) induces EMT and proliferation of GC cells, which leads to resistance of GC cells to CP therapy. By sponging miR-218, HOTTIP stimulates the expression of HMGA1 to trigger EMT (E-cadherin downregulation, and N-cadherin, and vimentin upregulation). By downregulating HOTTIP, the sensitivity of GC cells to CP was restored [213].

### 6.3. Forkhead Box Protein O1 (FOXO1) Signaling Pathway

FOXO1 is a tumor suppressor protein involved in inhibiting the migration and proliferation of cancer cells [214,215]. Prior studies have shown the involvement of FOXO1 in chemosensitivity [216]. In a newly published article by Li and colleagues, the role of FOXO1 in sensitizing nasopharyngeal carcinoma (NPC) cells to CP therapy was investigated mechanistically. In general, FOXO1 upregulates the expression of miR-200b by ZEB1 induction via the GSK-3β/β-catenin/TCF4 axis. This significantly sensitizes cancer cells to CP therapy by suppressing the EMT mechanism. MYH9 downregulation by FOXO1 also contributes to EMT inhibition [217]. This axis is important for the anti-tumor activity of cinobufotalin (CB). This substance is derived from toad venom and has shown a high anti-tumor activity [218]. CB stimulates the sensitivity of NPC cells to CP therapy by activating FOXO1 and subsequently inhibiting MYH9, which leads to a reduction in cancer rigidity and inhibition of EMT (E-cadherin upregulation, and N-cadherin downregulation) [217].

### 6.4. MicroRNAs

As it was mentioned in Section 3, miRs are involved in regulation of different biological mechanisms due to their capability in affecting various molecular pathways and mechanisms. Abnormal expression of miRs paves the way for the development of cancer [219,220]. MiR-146b is one of the main regulators of the innate and adaptive immune response [221]. The growing evidence has shed some light on the involvement of miR-146b in cancer, infection, and bone regeneration [222,223,224]. It appears that the effect of miR-146b on protein tyrosine phosphatase 1B (PTP1B) is important in CP therapy. PTP1B is involved in the development of cancer, and there are controversial data on its inhibitory or stimulatory effect in cancer [225,226]. In lung adenocarcinoma cells that are resistant to CP chemotherapy, increasing the expression of miR-146b can be considered a promising strategy. The upregulation of miR-146b remarkably reduces the expression of PTP1B. The in vitro and in vivo experiments confirm the reduced EMT concentration as a consequence of PTP1B downregulation by miR-146b, so that the expression of E-cadherin is upregulated, while the expression of N-cadherin decreases. This shows that EMT prevention by miR-146b/PTP1B axis can sensitize lung adenocarcinoma cells to CP therapy [227].

The miR-363 can act as an onco-suppressor miR. Its downregulation is associated with malignant and aggressive behavior of cancer cells [228]. The miR-363 is able to suppress myeloid cell leukemia 1 (Mcl-1), which leads to reduced proliferation and invasion of laryngeal cancer cells [90]. In view of the inhibitory effect of miR-363 on the malignancy of cancer cells, the lncRNA NNT-AS1 decreases the expression of this miR to increase the migration and invasion of GC cells [229]. In ovarian carcinoma cells resistant to CP, the increase in expression of miR-363 has an inverse relationship to screw expression. Downregulation of the Snail suppresses the EMT mechanism, which leads to the sensitivity of ovarian cancer cells to chemotherapy [230].

Inhibition of miR-200c by CD44 contributes to ZEB1 upregulation, leading to increased migration and invasion of cancer cells and CP resistance [231]. MiR-139-5p is an onco-suppressor miR that is able to reduce invasion, migration, and proliferation of tumor cells and to induce apoptotic cell death. The reduced expression of miR-139-56p is also associated with a poor prognosis in cancer patients [232,233,234,235]. In NPC cells, miR-139-5p suppresses EMT in order to significantly reduce the migration and invasion tumor cells, which leads to the sensitivity of tumor cells to CP chemotherapy [236].

### 6.5. PI3K/Akt Signaling Pathway

It is worth mentioning that a specific population of tumor cells known as cancer stem cells (CSCs) are involved in migration and metastasis [237,238,239,240]. These cells are also involved in cancer recurrences, and studies, both in vivo and in clinical trials, have shown the inadequacy of anti-tumor drugs in completely eliminating CSCs [241]. The relationship between CSCs and the PI3K/Akt/mTOR axis is important in cancer therapy. The Akt signaling pathway is involved in cell survival, metabolism, and differentiation [242,243,244,245,246]. It has been shown that mTOR is able to stimulate Akt in Ser^473^ [247]. In particular, it is believed that phosphorylation of Akt by mTOR contributes to tumor development, so that phosphorylated Akt inhibits GSK-3β [248,249]. The activity of GSK-3β is crucial for the increase of E-cadherin levels by cytoplasmic translocation of the cochlea [250]. The degradation of GSK-3β by Akt leads to stimulation of EMT [251,252]. This axis is remarkable in providing CP resistance in non-small cell lung carcinoma (NSCLC). Aspirin can be used to interrupt this axis and sensitize cancer cells to CP chemotherapy. Aspirin administration reduces the expression of the mTOR pathway to suppress Akt by dephosphorylation in Ser^473^. This stabilizes and induces GSK-3β to inhibit Snail and β-catenin nuclear translocation, which leads to inhibition of the EMT mechanism. Suppressed EMT sensitizes NSCLC cells to CP-mediated apoptosis [253].

### 6.6. Wnt Signaling Pathway

The Wnt signaling pathway is an important molecular signaling pathway with the ability to regulate biological processes such as apoptosis and differentiation [254,255,256,257]. A number of studies have shown that the Wnt signaling pathway contributes to increased invasion and malignancy of tumor cells via EMT induction [258,259,260]. The inhibition of Wnt by onco-suppressor factor Numb suppresses EMT in cancer cells, thereby illustrating the role of Wnt as an upstream mediator of EMT [261]. In addition, EMT induction by Wnt can elevate proliferation of cancer cells, and triggers their resistance into apoptosis [262]. On the other hand, the Wnt signaling pathway can be considered a secondary target of miRs (33a-5p) and lncRNAs (JPX) [263,264]. An increase in resistance to CP therapy has been observed in ovarian cancer cells. It has been proposed that miR-338-3p undergoes downward regulation in ovarian cancer cells and tissues. An increased expression of miR-338-3p sensitizes these tumor cells to CP therapy. Investigation of the molecular signaling pathways has shown that the overexpression of miR-338-3p can attenuate the Wnt2B signaling pathway for inhibiting EMT-mediated CP resistance [265]. Suppression of the Wnt signaling pathway is also important for the sensitization of malignant mesothelioma (MM) cells for CP therapy [266]. Epstein–Barr virus (EBV) infection is responsible for the development of NPC cells. EBV-miR-BART22 contributes to the resistance of NPC cells to chemotherapy. Mechanistically, EBV-miR-BART22 increases the expression of MYH9 by aligning with the PI3K/Akt/c-Jun axis. Then MYH9 induces the degradation of GSK-3β to mediate nuclear translocation of β-catenin, which leads to stimulation of the EMT mechanism and CP resistance. The interruption of this signaling pathway is therefore important for the inhibition of the EMT mechanism and the suppression of CP resistance. CB administration intervenes in the MYH9/GSK-3β/β−catenin axis by activating MAP2K4. This leads to inhibition of the EMT mechanism and sensitization of cancer cells to CP chemotherapy [267].

### 6.7. Long Non-Coding RNAs

The HOXA-AS3 gene is a member of the homeobox (HOX) family located on chromosome 7p15. It has been reported that this gene may be involved in the progression and metastasis of tumor cells [268,269,270]. It seems that the homeobox A3 (HOXA3) has a modulating influence on the EMT mechanism [271]. In NSCLC cells that are resistant to CP chemotherapy, the lncRNA HOXA-AS3 increases to inhibit HOXA3, leading to CP resistance. Inhibition of the lncRNA HOXA-AS3 increases the expression of HOXA3. Over-expressed HOXA3 inhibits EMT by increasing E-cadherin and reducing vimentin, resulting in the sensitivity of NSCLC cells to CP therapy [50]. The same applies to ovarian cancer cells. The LncRNA H19 increases the migration and malignancy of tumor cells by stimulating EMT, which makes these tumor cells resistant to CP therapy. Inhibition of the lncRNA H19 sensitizes ovarian cancer cells to CP-induced apoptosis by EMT inactivation [76]. These studies show that lncRNAs are involved in CP resistance by EMT induction.

The lncRNA urogenital carcinoma antigen 1 (UCA1) is another factor whose role in the resistance of NSCLC cells to CP therapy has been investigated. The lncRNA UCA1 has been considered as an oncogenic factor. It is believed that the lncRNA UCA1 induces the migration and invasion of thyroid cancer cells via miR-497-3p downregulation [272]. On the other hand, the lncRNA UCA1 reduces the expression of miR-654-5p to upregulate the expression of salt-inducible kinase 2 (SIK2), which leads to the development of PTX resistance [273]. In addition, other lncRNAs such as GAS8-AS1 can inhibit the lncRNA UCA1 to suppress the invasion of osteosarcoma cells [274]. The lncRNA UCA1 increases levels of N-cadherin, vimentin, and Snail, while decreasing levels of E-cadherin to induce the EMT mechanism, resulting in resistance of NSCLC cells to CP therapy [275]. Therefore, knock-out or knock-down of the lncRNA UCA1 and forkhead box protein C2 (FOXC2) can be considered as potential strategies for sensitizing NSCLC cells to CP chemotherapy.

### 6.8. Nuclear Factor-κB (NF-ĸB) Signaling Pathway

Nuclear factor-kappaB (NF-κB) is a transcription factor associated with pathological events including inflammation and cancer [112,273,276,277,278,279,280,281]. The IκB kinase (IKK) is an upstream mediator of NF-κB that is capable of regulating the proliferation and migration of cancer cells [282,283,284,285,286,287,288]. IKK expression is thought to be associated with higher levels of N-cadherin and EMT activation, which subsequently induces resistance of head and neck cancer cells to CP therapy. Suppression of IKK activity can attenuate EMT, which mediates sensitization of head and neck cancer cells to CP therapy [289].

### 6.9. Other Molecular Signaling Pathways

The forkhead box protein C2 (FOXC2) is one of the most important members of the forkhead box (FOX) family [249]. A large number of recently published articles have demonstrated the role of FOXC2 in improving the proliferation of cancer cells [290,291,292]. The expression of FOXC2 is believed to be associated with drug resistance and increased progression of melanoma cells [293]. This transcription factor significantly increases metastasis and progression of prostate cancer cells via the EMT mechanism [294,295]. Protein kinase C (PKC) also increases the expression of FOXC2 to inhibit p-120 catenin, leading to the migration and invasion of breast cancer cells [296]. The relationship between FOXC2 and EMT is of considerable importance in NSCLC cells. In A549 cells, FOXC2 increases Snail levels by Akt/GSK-3β upregulation. As a consequence of elevated Snail levels, EMT mechanism can be induced to mediate resistance of NSCLC cells to CP therapy [297].

Chrysotobibenzyl is a derivative of *Dendrobium pulchellum* with inhibitory activities against lung cancer cells [298]. The administration of chrysotobibenzyl is beneficial in alleviating CP resistance. It appears that chrysotobibenzyl inhibits EMT by reducing vimentin, Snail, and Slug to sensitize lung cancer cells to CP-induced apoptosis [299]. These studies show that different molecular signaling pathways may be involved in CP resistance and their identification is of considerable importance for the inhibition of resistance to CP therapy [300].

Vascular endothelial growth factor (VEGF) and programmed death ligand 1 (PD-L1) can contribute to increased migration and malignancy of cancer cells. The novel strategies are based on the inhibition of VEGF and PD-L1 [263,301,302,303]. Bevacizumab and atezolizumab are able to suppress VEGF and PD-L1 and inhibit the progression of cancer cells, respectively [304,305,306,307]. The administration of atezolizumab and bevacizumab can inhibit VEGF and PD-L1 to suppress ovarian cancer malignancy and sensitize them to CP therapy. It appears that the inhibition of VEGF and PD-L1 and the subsequent downregulation of the EMT mechanism are involved in sensitizing ovarian cancer cells to CP therapy [121].

p53 is an onco-suppressor transcription factor, the inactivation and/or mutation of which has been reported in various types of cancer [308,309]. On the contrary, it has been suggested that the mouse double minute 2 homologue (MDM2) is involved in p53 inhibition by mediating its proteosomal degradation [310]. Recently published articles have shown that inhibitors of MDM2 are beneficial in stimulating p53 and suppressing the malignancy of cancer cells [133,311]. In lung cancer cells, zinc finger CCHC-type containing 10 (ZCCHC10) can inhibit MDM2 to stimulate and enhance the expression of p53. Inhibition of p53 degradation leads to upregulation of the epithelial marker E-cadherin and downregulation of Snail and Slug as mesenchymal markers; this leads to suppression of EMT and sensitization of cancer cells to CP therapy [312].

Norcantharidin (NCTD) is a demethylated form of cantharidin isolated from the bladder beetle [313]. This agent has shown significant anti-tumor effects. The administration of NCTD is beneficial in suppressing the invasion of bladder cancer by inhibiting the DNA damage response (DDR) via cdc6 degradation [314]. NCTD is also beneficial in sensitizing breast cancer cells to tamoxifen chemotherapy by upregulating miR-873 and CDK3 inhibition [159]. Due to the significant anti-tumor activity of NCTD, it can sensitize cancer cells to CP chemotherapy. Administration of NCTD is associated with downregulation of YAP1 and its downstream mediators, connective tissue growth factor (CTGF), and cysteine-rich angiogenic inducer 61 (CYR61). This leads to inhibition of EMT and sensitization of NSCLC cells to CP chemotherapy [315].

It is obvious that Twist is an EMT marker that undergoes upregulation during cancer metastasis [314,316,317]. It is worth mentioning that Twist and EMT are able to regulate the resistance of cancer cells to chemotherapy [318,319]. Twist induces EMT by increasing N-cadherin and vimentin levels and decreasing E-cadherin levels [320,321]. Inhibition of Twist is believed to significantly reduce the migration and invasion of cancer cells [316,322]. In ovarian cancer cells, Twist activates the EMT mechanism to mediate CP chemoresistance. Suppression of Twist expression using siRNA-loaded hyaluronic acid-conjugated nanoparticles inhibits the EMT mechanism, which leads to sensitization of ovarian cancer cells to CP chemotherapy [323]. Programmed cell death 4 (PDCD4) is an important regulator of cell proliferation and apoptosis. PDCD4 is an onco-suppressor factor that inhibits cancer metastasis via EMT downregulation [232]. A number of processes such as autophagy can degrade PDCD4 to ensure the proliferation and malignancy of tumor cells [324].

The carcinogenic inhibitor of protein phosphatase 2A (CIP2A) is upregulated in malignant human diseases and is associated with a poor prognosis [69]. CIP2A contributes to increased metastasis of cancer cells by suppressing protein phosphatase 2A (PP2A) [325,326]. CIP2A is able to target molecular signaling pathways such as MEK and ERK in the activation of EMT [327]. There is growing evidence that CIP2A induces phosphorylation of Akt and subsequently mTOR to mediate CP resistance [297,328,329]. Targeting CIP2A/Akt/mTOR is therefore beneficial in preventing resistance to CP. Polyphyllin I (PPI) and polyphyllin VII (PPVII) are two bioactive components of Parisian polyphylla (PRS) that display substantial anti-tumor activities [330,331]. Administration of PPI and PPVII significantly inhibits the CIP2A/Akt/mTOR axis to attenuate EMT, resulting in sensitivity of NSCLC cells to CP chemotherapy [332].

It appears that in CP-resistant bladder cancer cells, increasing the expression of PDCD4 is important. The upregulation of PDCD4 is associated with the inhibition of the JNK/c-Jun signaling pathway to suppress EMT and sensitize bladder cancer cells to CP chemotherapy [333]. Considering the above evidence, a variety of signaling networks are involved in CP resistance by EMT induction, and studies have investigated the ability of various compounds and molecular pathways to inhibit the EMT mechanism that may sensitize cancer cells to chemotherapy.

## 7. Possible Pathways for Further Targeting

### 7.1. PI3k/Akt Signaling Pathway

PI3K/Akt signaling pathway regulates apoptosis, differentiation, metastasis, and drug resistance. It has been demonstrated that PI3K/Akt pathway, as an oncogene pathway, can elevate the invasion and migration of cancer cells by targeting EMT [334,335,336]. It has been reported that this pathway is of importance in CP resistance via EMT stimulation. On the other hand, miRs have been suggested to be involved in cancer invasion and EMT regulation [337,338]. In NPC cells resistant to CP chemotherapy, overexpressed miR-205-5p diminishes the expression of tumor suppressor factor, PTEN, by upregulation of PI3K/Akt signaling pathway, leading to the activation of EMT and resistance to CP chemotherapy [339]. The miR-205-5p/PI3K/Akt/PTEN can be targeted in future studies for inhibition of resistance to CP therapy.

### 7.2. Laminin Subunit Beta-3

The laminin subunit beta-3 (LAMB3) is an oncogenic factor capable of enhancing the proliferation and migration of pancreatic cancer cells via stimulation of PI3K/Akt signaling pathway [338]. It appears that LAMB3 induces EMT mechanism through MET/Akt activation, leading to an increased migration and malignancy of thyroid cancer cells [340]. Accumulating data show the stimulatory effect of LAMB3 on the viability and growth of tumor cells [341]. A similar phenomenon occurs in head and neck squamous cell carcinoma. It has been found that LAMB3 activates EMT mechanism through vimentin and Slug upregulation to reduce the CP efficacy in elimination of cancer cells. Suppressing the expression of LAMB3 diminishes the metastasis of tumor cells and sensitizes them to the cytotoxicity of CP therapy [338].

### 7.3. ZEB Proteins

The zinc-finger E-box binding homeobox (ZEB) family includes two major components: ZEB1 and ZEB2 [342,343]. The accumulating data demonstrate that ZEB1 and ZEB2 proteins are able to enhance the invasion and migration of cancer cells [344]. It has been shown that these proteins can induce EMT by E-cadherin downregulation and N-cadherin upregulation [345]. So, in respect to the involvement of EMT in resistance to CP, there is a relationship between ZEB proteins and CP resistance. On the other hand, excision repair cross-complementary gene 1 (ERCC1) and ATP-binding cassette subfamily G member 2 (ABCG2) can contribute to the chemoresistance [139,346,347]. It has been reported that UBE2C enhances the expressions of ZEB1 and ZEB2 to upregulate ZBCG2 and ERCC1. This axis results in EMT activation and enhanced invasion and malignancy of NSCLC cells. Then, these cancer cells with high proliferation and migration ability can induce resistance against CP therapy [348].

### 7.4. Long Non-Coding RNAs

The lncRNA homeobox A11 antisense RNA (HOXA11-AS) is suggested to be involved in elevating the migration and invasion of GC cells by miR-148a downregulation and subsequent activation of Wnt1/β-catenin signaling pathway [349]. In prostate cancer cells, HOXA11-AS induces actinin alpha 4 (ACTN4) expression through miR-518b sponging, leading to enhanced proliferation and migration of tumor cells [156]. Overall, the studies are in agreement with the role of lncRNA HOXA11-AS in tumorigenesis as well as in poor prognosis [96,350]. The relationship between lncRNA HOXA11-AS and CP resistance in lung adenocarcinoma (LUAD) cells is of importance, so that HOXA11-AS enhances the expression of STAT3 though miR-454-3p sponging. The HOXA11-AS/miR-454-3p/STAT3 axis leads to EMT activation and resistance of LUAD cells in CP therapy [351].

### 7.5. Targeting Drug Transporters

One of the reasons underlying the induction of chemoresistance is the enhanced activity and expression of drug transporters [352]. Accumulating data demonstrate that tumor cells are able to enhance expression of various transporters to reduce accumulation of chemotherapeutic agents and prevent their cellular uptake [353]. In respect to the adverse effects of chemotherapeutic agents, and some other unexpected outcomes, it is about impossible to administer high doses of a certain chemotherapeutic agent. As a consequence, novel strategies should be considered in targeting the drug transporters to sensitize cancer cells to chemotherapeutic agents via enhancing their cellular internalization [354]. The ATP-binding cassette (ABC) transporters are suggested to mediate chemoresistance via exporting chemotherapeutic drugs out of cells [355]. To date, up to 48 ABC transporters have been identified in humans. The P-glycoprotein (P-gp) is a key member of ABC transporters, known as ABCB1 [356]. This transmembrane glycoprotein has a molecular weight of 170 kDa consisting of two membrane spinning domains (MSDs) and two nucleotide binding domains (NBDs) that can be used for ATP binding [357,358]. The P-gp is expressed in a number of barriers including the blood–brain barrier (BBB), blood–testis barrier (BTB), and blood–cerebrospinal fluid barrier to modulate absorption and excretion of xenobiotics [359]. Although normal activity of P-gp is vital in physiological conditions, its upregulation is an increasing impediment in cancer therapy, since cancer cells are able to trigger chemoresistance by enhancing expression of P-gp [360,361]. As mentioned earlier, administration of CP has been correlated with stimulation of EMT. It has been found that in lung cancer cells exposed to CP, an increase in EMT concentration occurred by the TGF-β signaling pathway. The overexpression of EMT paves the road for induction of P-gp that subsequently exports CP out of tumor cells, thereby leading to chemoresistance [362]. Recently, it was reported that CP-resistant cancer cells that underwent EMT demonstrated an increase in N-cadherin and spindle-shaped cells. Although this study did not explore the relationship between EMT and expression of P-gp, it seems that EMT induction substantially enhances migration and metastasis of cancer cells. This stimulation in malignant behavior of cancer cells can facilitate an overexpression of P-gp, and resistance of cancer cells to CP therapy [363].

### 7.6. Other Molecular Signaling Pathways

Psoriasin is a member of the calcium-binding EF-hand proteins with a molecular weight of 11.4 kDa. This oncogene protein is found in cells or can be secreted outside the cells [364,365,366,367]. In the field of cancer, psoriasin overexpression has been related to the poor prognosis of patients [263]. This is due to the stimulatory impact of psoriasin on the migration, invasion, and malignancy of cancer cells [368]. In vitro experiments on GC cells show that psoriasin upregulation can reduce ERK signaling pathway to activate EMT and partially mediate the resistance of tumor cells to CP chemotherapy [369]. It has been found that diverse signaling cascades contribute to the CP resistance of tumor cells. For instance, downregulation of Raf kinase inhibitor protein (RKIP) by miR-27a mediates the positive impacts on the proliferation and EMT induction in liver cancer cells. The malignant tumor cells can then acquire resistance to CP chemotherapy [369]. Human HLA-F adjacent transcript 10 (FAT10) is considered as an oncogenic factor with the capability of regulation of apoptosis and differentiation [334,370].

Targeting UBE2C can disrupt the aforementioned axis to sensitize NSCLC cells in CP therapy. The eukaryotic initiation factor 5A2 (elF5A2) is involved in modulation of vital biological processes such as proliferation, differentiation, and apoptosis [371,372]. Various studies have shed light on the involvement of elF5A2 in the migration and metastasis of cancer cells [373,374,375,376]. In GC cells, elF5A2 induces CP resistance by EMT stimulation through enhancing vimentin and N-cadherin levels and decreasing E-cadherin and β-catenin levels [377].

Deubiquitinating enzymes (DUBs) can inhibit the degradation of proteins through prevention of ubiquitination [378]. The abnormal expression of DUBs occurs in tumor malignancy and EMT [379,380]. The ubiquitin specific peptidase 37 (USP37) is a kind of DUB containing 979 amino acids with an oncogene role [381,382]. It appears that USP37 can also facilitate G1/S transition [383,384]. A newly published article demonstrated that USP37 stabilizes Snail protein via deubiquitination to enhance the migration and metastasis of lung cancer cells [385]. On the contrary, Hedgehog (Hh) pathway has been found to be involved in the migration of breast cancer cells via EMT mechanism [386]. The USP37 can stimulate Hh signaling pathway through Smo and Gli-1 upregulation. This axis leads to EMT activation to induce CP resistance in breast cancer stem cells [387]. The ATM is involved in resistance of chemotherapy in cancer cells. It has been reported that oncogenic JAK/STAT3 signaling pathway activation can trigger ATM-mediated EMT [388,389,390,391,392,393]. On the other hand, administration of CP induces EMT through ATM upregulation. In addition, PD-L1 can elevate the progression and metastasis of cancer cells through EMT [394,395]. In lung cancer cells, ATM induces JAK/STAT3 signaling pathway through PD-L1 upregulation to activate EMT, thus resulting in resistance of cancer cells in CP therapy [396]. Interestingly, knock-out of ATM can reverse this axis to enhance the sensitivity of cancer cells to CP therapy.

Visfatin released by adipocytes and inflammatory cells is involved in various biological mechanisms such as angiogenesis, inflammation, and cell growth [397,398]. In recent years, much attention has been directed towards visfatin as a biomarker for diagnosis of cancer [399]. Visfatin induces growth differentiation factor 15 (GDF15)/Akt axis to elevate the progression and proliferation of tumor cells [322]. These studies highlight the fact that visfatin can accelerate the progression of tumor cells [400]. Visfatin follows an interesting route in resistance of cancer cells. In osteosarcoma cells, visfatin enhances the expression of Snail through stimulation of hypoxia-inducible factor-α (HIF-α) transcription, while visfatin has no effect on the expression of ZEB1 and elevates its stability via ATM induction. Consequently, induced ZEB1 and Snail can mediate EMT activation, leading to the repression of osteosarcoma cells to CP chemotherapy [401]. Cyclooxygenase-2 (COX-2) is another important protein and its role in cancer has been extensively explored. It is worth mentioning that suppressing COX-2 can inhibit the invasion of cancer cells [402]. In addition, oncogenic factors such as BPTF enhances the expression of COX-2 to stimulate cancer cell growth and proliferation [403]. It has been found that COX-2 induces Akt signaling pathway to activate EMT mechanism. The EMT elevates the malignancy and invasion of NSCLC cells and induces resistance to CP therapy [404]. Overall, inhibition of visfatin and COX-2 can be advantageous in making cancer cells sensitive to CP therapy by promoting EMT downregulation.

Pyruvate dehydrogenase complex (PDC) is involved in cellular respiration through converting pyruvate into acetyl-coA. Pyruvate dehydrogenase kinase 1 (PDK1) is a key member of glycolytic enzymes that disrupts cellular respiration via PDC phosphorylation. This leads to the enhanced level of lactate in cytosol and inhibition of pyruvate oxidation in mitochondria [405]. Accumulating data demonstrate that overexpression of PDK1 can occur in various cancers [406,407]. Suppressing PDK1 has resulted in an increase in ROS generation and induction of apoptosis in cancer cells [39,408,409]. On the contrary, the epidermal growth factor receptor (EGFR) plays a significant role in development of cancer. It appears that in cutaneous squamous cell carcinoma, EGFR activates NF-κB signaling pathway to elevate the progression and malignancy of cancer cells [410]. Furthermore, the capability of EGFR in stimulation of EMT has been shown [411,412]. In the case of CP, PDK1 upregulates the expression of EGFR and inhibition of PDK1/EGFR axis can suppress EMT and malignancy of tumor cells. This results in greater sensitivity of ovarian cancer cells to chemotherapy [251]. In addition to the EGFR, insulin-like growth factor 1 (IGF1-R) contributes to the malignancy and invasion of tumor cells by stimulation of EMT mechanism. For doing this, IGF1-R can affect a variety of signaling cascades including STAT3/Akt and PI3K/Akt [154,413]. In ovarian cancer cells, IGF1-R undergoes upregulation as a consequence of miR-1294 downregulation. The overexpressed IGF1-R enhances the migration of cancer cells through EMT induction. It is worth mentioning that IGF1-R-mediated EMT may be involved in resistance of ovarian cancer cells in response to CP therapy [351]. These studies highlight the fact that elevating the expression of miR-1294 and PDK1 inhibition may be beneficial in sensitizing ovarian cancer cells in CP therapy.

As mentioned earlier, Snail is a transcription factor involved in regulation of EMT. Understanding the link between Snail and eukaryotic translation initiation factor 4E (elF4E) is vital for inhibiting CP resistance in NPC cells. The abnormal expression of elF4E occurs in a variety of cancers [414,415,416,417]. The elF4E upregulation is associated with malignant transformation of normal cells and enhanced invasion of tumor cells [417,418]. Suppressing the nuclear translocation of β-catenin via targeting elF4E can sensitize NPC cells to apoptotic cell death. In NPC cells and tissues, the expression of elF4E undergoes upregulation to provide the nuclear translocation of Snail. Thereafter, EMT mechanism can be activated to enhance the malignancy and invasion of NPC cells and make them resistant to CP chemotherapy [419]. This connection between elF4E and Snail can be considered as a promising candidate for sensitizing tumor cells to CP therapy, and further targeting these proteins, both pharmacologically and genetically, can pave the road for suppressing resistance to CP. It is worth mentioning that in cancer cells resistant to CP therapy, the expression of molecules involved in EMT such as N-cadherin has been found to be elevated. As a result of EMT induction, the morphology of cells can undergo transformation from round to spindle-shaped [363].

With respect to the role of Wnt signaling pathway in EMT induction, this pathway may also confer resistance to CP therapy. B-cell lymphoma 9 (BCL9) is an oncogenic factor and its upregulation is associated with poor prognosis of patients with cancer, low incidence of apoptosis in cancer cells, and increased malignancy [47,97,410]. In NSCLC cells, BCL9 stimulates the nuclear translocation of β-catenin to activate EMT mechanism and induce resistance of tumor cells to CP chemotherapy [351].

**Figure 2:**
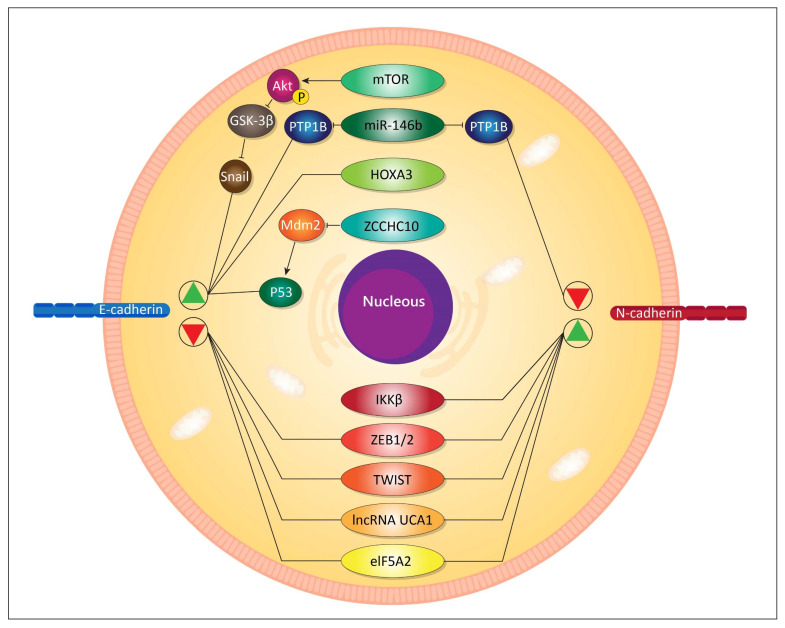
A summary of selected molecular pathways regulating epithelial–mesenchymal transition (EMT) and their possible involvement in resistance to CP therapy. mTOR, mammalian target of rapamycin; Akt, protein kinase B; GSK-3β, glycogensynthase kinase-3β; miR, microRNA; PTP1B, protein tyrosine phosphatase 1B; HOXA3, homeobox A3; Mdm2, mouse double minute 2 homolog; IKKβ, Inhibitor of NF-κB kinase subunit β; ZEB, zinc-finger E-box binding homeobox; lncRNA, long non-coding RNA; UCA1, urogenital carcinoma antigen 1; elF5A2, eukaryotic initiation factor 5A2.

In osteosarcoma cells, FAT10 induces the ubiquitin-mediated degradation of YAP1 to elevate the viability and growth of tumor cells [152]. Investigation of the relationship between FAT10 and CP resistance can pave the road for the effective treatment of bladder cancer. It has been reported that overexpression of FAT10 induces EMT mechanism to elevate the malignancy of cancer cells and reduce the efficacy of CP chemotherapy [369]. This was the first study that has examined the relationship between FAT10 and EMT and its role in the development of resistance to CP therapy. However, further studies should focus on inhibition of FAT10, both pharmacologically and genetically. H2A histone family member Z (H2A.Z) is a potential regulator of proliferation, differentiation, DNA replication, chromosome segregation, and cell cycle [420,421,422,423,424]. Thus, targeting H2A.Z may influence the potential of CP in chemotherapy through its impact on the malignancy of cancer cells. It appears that H2A.Z can diminish the sensitivity of cancer cells to CP chemotherapy by induction of EMT and enhancing the progression of tumor cells [425]. Overall, a number of pathways can regulate the EMT process and contribute to chemoresistance (Figure 2).

## 8. Conclusions and Remarks

In the present review, we analyzed the involvement of the EMT mechanism in the induction of resistance to CP. As mentioned above, CP is an effective anti-tumor agent that negatively affects the viability and proliferation of tumor cells. Recent studies have shown the great potential of cancer cells to develop CP resistance to chemotherapy. It appears that EMT is one of the most important mechanisms associated with resistance to this drug. To fully describe the role of EMT in chemoresistance, we divided the article into three different sections. In the first section we have shown that the administration of CP is associated with EMT and tumor cell resistance. It was reported that both short and long treatment with CP can induce resistance. Autophagy, NF-κB, CAMs, and ATM are induced by CP to induce EMT and reduce the efficacy of chemotherapy, while downregulation of SLFN1 can also mediate resistance. These studies show that CP itself is capable of inducing EMT and promoting resistance in cancer cells and the above mechanisms and signaling pathways may be involved in this process.

It is worth mentioning that lncRNAs such as HOTTIP, HOXA-AS3, H19, and UCA1 are involved in resistance by targeting the EMT mechanism, and studies have focused on restoring the expression of these lncRNAs to increase CP cytotoxicity. Another possible strategy for inhibiting EMT-mediated resistance is miR upregulation/downregulation. In this case, studies have investigated the role of miR-218, -200b, -146b, -338-3p, -363, -139-5p, and so on. Overall, it has been concluded that manipulation of miR expression may pave the way for inhibition of EMT-mediated resistance to CP. In particular, in addition to lncRNAs and miRs, other molecular pathways such as PI3K/Akt, NF-κB, FOXO1, Wnt/catenin, mTOR, etc., can also influence EMT and chemoresistance. In the last section we presented a variety of molecular pathways that contribute to EMT-mediated resistance to CP and which can be specifically investigated in further studies.

Taking all these aspects into account, a number of complex and dynamic molecular pathways that contribute to resistance to therapy has been summarized here. These pathways may synergistically induce EMT and promote chemoresistance. In view of the role of more than one molecular pathway in EMT-mediated resistance, the focus in enhancing CP chemotherapy should be on targeting different oncogenic pathways. Finally, it should be mentioned that the studies were based on in vitro and in vivo findings and the lack of clinical trials shows that there is still a long way to go in developing strategies to suppress EMT-mediated CP resistance. In addition, disseminated tumor cells (DTCs) are also suggested to be viable after several years. They may reactivate and regrow after a long period of time and are considered as a vital reason for the recurrence of cancer. Prior studies have shown that existence of dormant cells ensures metastasis of cancer cells and is correlated with poor prognosis [483,484,485,486]. To date, there is no experiment about the relationship between CP, EMT, and dormant cells. However, with respect to the significant role of dormant cells in metastasis and recurrence, this can be evaluated in further studies.

## Figures and Tables

**Table 1:** The involvement of diverse molecular pathways in EMT-mediated resistance to CP therapy.

Cancer Type	Signaling Axis	Effect on EMT	Results	Refs
Lung cancer	EMT/PD-L1/PD-1	-	The CP-resistant lung cancer cells activate EMT to induce the expression of PD-L1/PD-1, ensuring their survival and resistance to CP therapy. Anti-PD-1 or anti-PD-L1 therapies may lead to the sensitivity of cancer cells in CP chemotherapy	[426]
Lung cancer	TGF-β1/EMT	Induction	TGF-β1 is able to stimulate EMT via enhancing N-cadherin and vimentin and decreasing E-cadherin. In addition, drug-resistant proteins such as ERCC1 and p-glycoprotein undergo upregulation. Inhibition of TGF-β1 enhances the sensitivity of cancer cells in CP therapy	[362]
Squamous cell carcinoma	SOX8/Wnt-β-catenin/EMT	Induction	SOX8 induces EMT through Frizzled-7-mediated Wnt signaling pathway to diminish the efficacy of CP chemotherapy	[427]
Lung cancer	MF-κB/EMT	Induction	The administration of ginsenoside Rg3 sensitizes cancer cells in CP chemotherapy by inhibition of NF-κB-induced EMT	[428]
Ovarian cancer	-	-	PD98059 induces EMT mechanism to reduce the sensitivity of cancer cells to CP chemotherapy	[429]
Breast cancer	NF-κB/EMT	Induction	Eugenol inhibits EMT through NF-κB downregulation, leading to the enhanced anti-tumor activity of CP	[430]
Cervical cancer	RIF1/EMT	Induction	RIF1 enhances the malignancy of cancer cells through EMT induction. Suppressing RIF1 sensitizes cancer cells in CP chemotherapy	[431]
Breast cancer	αvβ3 integrin/EMT FAK/PI3K/Akt/EMT	Induction	14, 15-EET trigger EMT through activation of FAK/PI3K/Akt and αvβ3 integrin to ensure the resistance of cancer cells in CP chemotherapy	[432]
Ovarian cancer	Notch3/SUSD2/EMT	Induction	The downstream mediator of Notch3, SUSD4 induces EMT to trigger the resistance of cancer cells in CP chemotherapy	[433]
Lung cancer	TGF-β1/EMT	Induction	Inhibition of TGF-β1 is associated with EMT downregulation and consequently, the sensitivity of cancer cells in CP chemotherapy	[434]
Ovarian cancer	MiR-20a/EMT	Induction	The miR-20a induces CP resistance in cancer cells through EMT induction	[435]
Non-small cell lung cancer	SLC39A4/EMT	Induction	The inhibition of SLC39A4 suppresses EMT and sensitizes cancer cells in CP chemotherapy	[436]
Nasopharyngeal carcinoma	Hippo/TAZ/EMT	Induction	The TAZ activation as a key gene of Hippo pathway induces EMT and triggers the resistance of cancer cells in CP chemotherapy	[437]
Colorectal cancer	hERG1/EMT	Induction	The hERG1 ion channels induce EMT to diminish the sensitivity of cancer cells in CP chemotherapy	[438]
Nasopharyngeal carcinoma	TIMELESS/EMT	Induction	Suppressing TIMELESS expression inhibits EMT to enhance the efficacy of CP chemotherapy	[439]
Ovarian cancer	MiR-30a/c-5p/DNMT1/EMT	Inhibition	The overexpressed miR-30a/c-5p inhibits EMT through DNMT1 downregulation, leading to the sensitivity of cancer cells in CP chemotherapy	[440]
Cervical cancer	iASPP/miR-20a/FBXL5/BTG3/EMT	Induction	The iASPP inhibits FBXL5/BTG3 axis through miR-20a upregulation. As a consequence, EMT mechanism occurs to ensure the resistance of cancer cells in CP chemotherapy	[441]
Nasopharyngeal carcinoma	NEDD4/EMT	Induction	Knock-out of NEDD4 inhibits EMT and induces mesenchymal–epithelial transition, resulting in sensitivity of cancer cells in CP chemotherapy	[442]
Oral tongue squamous cell cancer	MiR-15b/TRIM14/EMT	Inhibition	The upregulated miR-15b suppresses EMT via TRIM14 downregulation to induce mesenchymal –epithelial transition and sensitize cancer cells in CP chemotherapy	[443]
Lung adenocarcinoma	PI3K/Akt/NF-κB/EMT	Induction	The administration of baicalein inhibits the PI3K/Akt/NF-κB-mediated EMT to sensitize cancer cells in CP chemotherapy and reduce their malignancy and migration	[444]
Oral tongue squamous cell carcinoma	has-miR-485-5p/PAK1/ERCC1-YAP/EMT	Inhibition	The downstream mediators of PAK1 including ERCC1 and YAP are involved in induction of EMT and resistance of cancer cells in CP chemotherapy. The hsa-miR-485-5p disrupts the aforementioned axis to suppresses EMT and malignancy, and restore the sensitivity to CP treatment	[445]
Gastric cancer	TAZ/EMT	Induction	The overexpression of TAZ stimulates EMT to ensure the malignancy and invasion of cancer cells, leading to resistance in CP therapy	[446]
Non-small cell lung cancer	ARK5/EMT	Induction	The ARK5 triggers EMT to enhance the malignancy and metastasis of cancer cells. The silencing of ARK5 sensitizes cancer cells in CP chemotherapy through EMT inhibition	[447]
Non-small cell lung cancer	Aurora A/EMT	Induction	The downregulation of Aurora A inhibits EMT, leading to the sensitivity of cancer cells in CP chemotherapy	[448]
Ovarian cancer	HPIP/PI3K/Akt/EMT	Induction	HPIP elevates the malignancy and invasion of cancer cells by EMT induction via PI3K/Akt axis. This enhanced malignancy leads to the resistance in CP chemotherapy	[449]
Ovarian cancer	EMT/Akt	-	The EMT induces Akt activation to drive the resistance in CP chemotherapy	[450]
Non-small cell lung cancer	MiR-101/ROCK2/EMT	Inhibition	The miR-101 has a reverse relationship with ROCK2 to inhibit EMT and promote the sensitivity of cancer cells in CP chemotherapy	[451]
Non-small cell lung cancer	FASN/TGF-β1/FASN	Induction	A positive loop between FASN and TGF-β1 induces EMT and triggers CP resistance	[452]
Cervical cancer	MiR-25-3p/Sema4C/EMT	Inhibition	By inhibition of Sema4C, miR-25-3p suppresses EMT to inhibit cancer metastasis and malignancy and sensitize them in CP chemotherapy	[453]
Ovarian cancer	FOXC2/ERK/EMT FOXC2/Akt/GSK-3β/EMT	Induction	The FOXC2 activates EMT mechanism via ERK and Akt/GSK-3β signaling pathways to induce the resistance of cancer cells in CP chemotherapy	[454]
Lung cancer	KLF4/EMT	Inhibition	The upregulation of KLF4 inhibits EMT mechanism through enhancing Slug, Twist and vimentin levels, and reducing E-cadherin levels, resulting in decreased malignancy and induction of apoptosis by CP chemotherapy	[455]
Nasopharyngeal carcinoma	MiR-374a/PDCD4/CCND1/ PI3K/Akt/c-Jun/EMT	Inhibition	The miR-374a downregulates the expression of CCND1 by induction of tumor suppressor PDCD4. Then, a decrease occurs in PI3K/Akt/c-Jun signaling pathway to inhibit EMT, leading to the sensitivity of cancer cells in CP chemotherapy	[456]
Neuroblastoma	MYH9 ACTN4 ROCK1	Induction Induction Inhibition	Overexpressed MYH9 and ACTN4, and decreased expression of ROCK1, are involved in EMT and resistance of cancer cells in CP chemotherapy	[457]
Ovarian cancer	MiR-186/Twist1/EMT	Inhibition	The miR-186 inhibits EMT through Twist1 downregulation to drive the sensitivity of cancer cells in CP chemotherapy	[458]
Cervical cancer	URI/EMT	Induction	The silencing of URI inhibits EMT to sensitize cancer cells in CP chemotherapy	[459]
Ovarian cancer	MiR-496 MiR-485-5p MiR-152 Let-7g	Induction	These miRs are associated with induction of EMT and resistance of cancer cells in CP chemotherapy	[460]
Triple negative breast cancer	-	-	The administration of niclosamide inhibits EMT to enhance the induction of apoptotic cell death	[461]
Gastric cancer	HER2/Snail/EMT	Induction	The HER2 activates Snail/EMT axis to reduce the efficacy of CP chemotherapy	[462]
Lung cancer	-	-	Pemetrexed pretreatment inhibits EMT to sensitize cancer cells in CP chemotherapy	[463]
Gastric cancer	HIF-α/miR-421/EMT	Induction	The HIF-α-mediated miR-421 upregulation reduces E-cadherin levels, leading to the EMT stimulation and CP resistance	[464]
Lung adenocarcinoma	MiR-206/MET/PI3K/AKT/ mTOR/EMT	Inhibition	The inactivation of MET/PI3K/Akt/mTOR axis by miR-206 inhibits EMT and drives the sensitivity of cancer cells in CP chemotherapy	[465]
Nasopharyngeal carcinoma	MiR-10b/KLF4/Notch1/E-cadherin	Induction	The miR-10b upregulates the expression of Notch1 via KLF4 inhibition to reduce E-cadherin levels, leading to the stimulation of EMT and CP resistance	[466]
Gastric cancer	MiR-30a/EMT	Inhibition	The overexpressed miR-30a reduces EMT to sensitize cancer cells in CP chemotherapy	[467]
Non-small cell lung cancer	elF5A2/EMT	Induction	GC7 inhibits EMT through elF5A2 downregulation, resulting in sensitivity of cancer cells in CP chemotherapy	[468]
Laryngeal carcinoma cells	CK2α/EMT	Induction	Silencing of CK2α suppresses EMT to inhibit malignancy and resistance of cancer cells in CP chemotherapy	[469]
Lung adenocarcinoma	Cx43/EMT	Inhibition	Cx43 reduces the malignancy and invasion of cancer cells through EMT inhibition, leading to the sensitivity in CP chemotherapy	[470]
Non-small cell lung cancer	MiR-17 MiR-20a MiR-20b	Inhibition	These miRs are able to suppress EMT and sensitizing cancer cells in CP chemotherapy	[471]
Head and neck squamous cell carcinoma	SET/EMT	Induction	As an oncogenic factor, SET induces EMT to reduce the sensitivity of cancer cells in CP chemotherapy	[472]
Ovarian cancer	ERK/Snail/EMT	Induction	The administration of resveratrol diminishes the expression of ERK to inhibit Snail and EMT, leading to the sensitivity of cancer cells in CP chemotherapy	[473]
Head and neck squamous cell carcinoma	-	-	Benzyl isothiocyanate suppresses CP resistance through EMT downregulation	[474]
Non-small cell lung cancer	FBW7/EMT	Inhibition	The overexpression of FBW7 inhibits EMT to enhance the sensitivity of cancer cells in CP chemotherapy	[475]
Prostate cancer	MiR-205/autophagy/EMT	Inhibition	The overexpression of miR-205 inhibits autophagy to suppress EMT and sensitize cancer cells in CP chemotherapy	[476]
Lung cancer	SET/NDRG1/EMT	Induction	The inhibition of NDRG by SET triggers EMT, leading to the resistance of cancer cells in CP chemotherapy	[477]
Ovarian cancer	Akt/EMT NF-κB/EMT	Induction	Gold nanoparticles are able to inhibit Akt and NF-κB signaling pathways to suppress EMT and CP resistance	[478]
Hepatic cancer	PDCD5/TGF-β/EMT	Inhibition	By downregulation of TGF-β, PDCD5 inhibits EMT to sensitize cancer cells in CP chemotherapy	[479]
Atypical teratoid/rhabdoid tumor	STAT3/Snail/EMT	Induction	Inhibition of STAT3/Snail axis suppresses EMT and partially sensitizes cancer cells in CP chemotherapy	[480]
Lung adenocarcinoma	MiR-15b/PEBP4/EMT	Induction	By downregulation of PEBP4, miR-15b triggers EMT mechanism to mediate the resistance of cancer cells in CP chemotherapy	[481]
Bladder cancer	DOCK1/EMT	Induction	Silencing DOCK1 expression is associated with sensitivity of cancer cells to CP via EMT inhibition	[482]

## Abbreviations

EMT	Epithelial–mesenchymal transition
CP	Cisplatin
ROS	Reactive oxygen species
DOX	Docetaxel
NPs	Nanoparticles
AMPK	AMP-activated protein kinase
lncRNA	Long non-coding RNAs
TAMs	Tumor associated macrophages
GC	Gastric cancer
OSCC	Oral squamous cell carcinoma
Nrf2	Nuclear factor erythroid 2-related factor 2
SIRT1	Sirtuin 1
MSI2	Musashi RNA-binding protein 2
FHL	Four-and-half LIM domain
TGF	Tumor growth factor
YAP1	Yes-associated protein 1
TAZ1	Tazaffin
CRC	Colorectal cancer
UBE2O	Ubiquitin-conjugating enzyme E2O
mTOR	Mammalian target of rapamycin
TKI	Tyrosine kinase inhibitor
PTX	Paclitaxel
Cams	Classically activated macrophages
HCC	Hepatocellular carcinoma
CCL20	Chemokine ligand 20
CCR6	Chemokine receptor 6
EMP3	Epithelial membrane protein 3
NPC	Nasopharyngeal carcinoma
EBV	Epstein-Barr virus
PTP1B	Protein tyrosine phosphatase 1B
CSCs	Cancer stem cells
NSCLC	Non-small cell lung cancer
CIP2A	Cancerous inhibitor of protein phosphatase 2A
PP2A	Protein phosphatase 2A
PPI	Polyphyllin I
PPVII	Polyphyllin VII
PRS	*Paris polyphylla*
MM	Malignant mesothelioma
HOXA3	Homeobox A3
FOXC2	Forkhead box protein C2
FOX	Forkhead box
PKCα	Protein kinase Cα
UCA1	Urogenital carcinoma antigen 1
SIK2	Salt inducible kinase 2
VEGF	Vascular endothelial growth factor
PD-L1	Programmed death-ligand 1
MDM2	Mouse double minute 2 homolog
ZCCHC10	Zinc finger CCHC-type containing 10
NF-κB	Nuclear factor-κB
IKKβ	Inhibitor of NF-κB kinase subunit β
Mcl-1	Myeloid cell leukemia 1
NCTD	Norcantharidin
DDR	DNA damage response
CTGF	Connective tissue growth factor
CYR61	Cysteine rich angiogenic factor 61
PDCD4	Programmed cell death 4
elF4E	Eukaryotic translation initiation factor 4E
MDR1	Multidrug resistance protein 1
LAMB3	Laminin subunit β-3;
ZEB	Zinc-finger E-box binding homeobox
ERCC1	Excision repair cross-complementary gene 1
ABCG2	ATP-binding cassette subfamily G member 2
elF5A2	Eukaryotic initiation factor 5A2
DUBs	Deubiquitinating enzymes
USP37	Ubiquitin specific peptidase 37
Hh	Hedgehog
ATM	Ataxia telangiectasia mutated
GDF15	Growth differentiation factor 15
HIF-α,	Hypoxia inducible factor-α
COX-2	Cyclooxygenase-2
PDC	Pyruvate dehydrogenase complex
PDK1	Pyruvate dehydrogenase kinase 1
EGFR	Epidermal growth factor receptor
IGF1R	Insulin-like growth factor 1
HOXA11-AS	Homeobox A11 antisense RNA
ACTN4	Actinin alpha 4
LUAD	Lung adenocarcinoma
BCL9	B-cell lymphoma 9
RKIP	Raf kinase inhibitor protein
FAT10	HLA-F adjacent transcript 10
H2A Z	H2A histone family member Z
ABC	ATP-binding cassette
P-gp	P-glycoprotein
MSDs	Membrane spinning domains
NBDs	Nucleotide binding domains
BBB	Blood–brain barrier
BTB	Blood–testis barrier
